# Inhibition of inosine metabolism of the gut microbiota decreases testosterone secretion in the testis

**DOI:** 10.1128/msystems.00138-24

**Published:** 2024-03-12

**Authors:** Lei Tang, Xizhong Yang, Mengting Zhou, Lingxin Feng, Cuijie Ji, Jie Liang, Bei Zhang, Ruowu Shen, Luoyang Wang

**Affiliations:** 1 Department of Special Medicine, School of Basic Medicine, Qingdao University, Qingdao, China; 2 Department of Spine Surgery, Qingdao Haici Medical Group, Qingdao, China; 3 Department of Immunology, School of Basic Medicine, Qingdao University, Qingdao, China; 4 Department of Oncology, The Affiliated Hospital of Qingdao University, Qingdao, China; Universita degli Studi di Napoli Federico II, Naples, Italy

**Keywords:** antibiotic, gut microbiota, testosterone, colistin, inosine, *Akkermansia*, testis

## Abstract

Growing evidence indicates that gut microbiota is involved in the regulation of the host’s sex hormone levels, such as through interfering with the sex hormone metabolism in the intestine. However, if gut microbiota or its metabolites directly influence the sex hormone biosynthesis in the gonad remains largely unknown. Our previous study showed that colistin, as a narrow-spectrum antibiotic, can significantly downregulate the serum testosterone levels and thus enhance the antitumor efficiency of anti-PD-L1 in male mice; however, the underlying mechanism for the regulation of the host’s testosterone levels remains uninvestigated. In the present study, we analyzed the impact of colistin on the immune microenvironment of the testis as well as the composition and metabolism of gut microbiota in male mice. Our results showed that colistin has an impact on the immune microenvironment of the testis and can downregulate serum testosterone levels in male mice through inhibition of *Akkermansia*, leading to destroyed inosine metabolism. Supplement with inosine can restore testosterone secretion probably by prompting the recovery of the intestinal mucus barrier and the serum lipopolysaccharides levels. All these findings reveal a new pathway for the regulation of the host’s sex hormone levels by gut microbiota.

**IMPORTANCE:**

This study demonstrates that exposure to even narrow-spectrum antibiotics may affect the host’s testosterone levels by altering the gut microbiota and its metabolites. Our findings provide evidence that some specific gut bacteria have an impact on the sex hormone biosynthesis in the testis.

## INTRODUCTION

Immune checkpoint inhibitors (ICIs) have revolutionized tumor therapy and are being increasingly used for the treatment of numerous cancer types (1). However, the relatively low response rate to these agents restricts the number of patients able to achieve effective tumor control (2). Many factors, including tumor intrinsic properties, tumor microenvironment, and environmental factors, such as diet and drug use (3, 4), can affect the antitumor efficiency of ICIs. Recently, sex hormones have emerged as key players in tumor immunotherapy (5). Androgen signaling could induce immunosuppression in cancer by inhibiting the activity and stemness of CD8^+^ T cells (6, 7). Estrogen could stimulate melanoma growth in murine models by interfering with macrophage polarization via estrogen receptor α and inducing CD8^+^ T cell exhaustion and ICI resistance (8). In contrast, estrogen receptor β agonists could promote CD8^+^ T cell activation and enhance antitumor immunotherapy (9). Besides, the gut microbiota was also recognized as essential in regulating tumor immunotherapy in recent years (10–14).

Interestingly, recent evidence indicates the existence of the sex hormone-gut microbiome axis (15). Changes in sex hormone levels can alter the composition of gut microbiota (16, 17). In turn, the transfer of some specific bacteria can induce changes in the host’s sex hormone levels (18–21). Thus, modulation of gut microbiota may represent a new way to regulate the host’s sex hormone levels (15). Our previous study showed that oral colistin administration can significantly downregulate the testosterone levels of male mice and thus enhance the antitumor efficacy of anti-PD-L1 antibody (22). Similarly, it was reported that a cocktail of broad-spectrum antibiotics could decrease testosterone levels in male mice with surgical castration but not in sham-operated mice via disruption of androgen biosynthesis by gut microbiota (23). In contrast, colistin is a narrow-spectrum antibiotic against Gram-negative bacteria but can downregulate serum testosterone levels in male mice without castration (22). These findings hint that colistin may affect the host’s hormone metabolism in a different way. However, the underlying mechanisms remain uninvestigated.

Commensal gut microbiota may affect the host’s sex hormone levels in various ways. Both sex hormones and their hydroxylated metabolites can be further modified via conjugation with sulfate or glucuronide, mainly in the liver, and excreted in urine or via bile to the intestine (24, 25). The conjugated metabolites in the gut can then be reabsorbed through the enterohepatic circulation after being deconjugated by the gut microbial sulfatases or β-glucuronidase (26). Besides, the gut microbiota can also express some steroid-processing enzymes that can directly metabolize steroid hormones (23, 27). Thus, the gut serves as an essential source of sex hormones. However, if gut microbiota or its metabolites directly influence the sex hormone biosynthesis in the gonad remains largely unknown.

In the present study, we analyzed the impact of colistin on the immune cell infiltration in the testis and the composition and metabolism of gut microbiota in male mice. Our results showed that colistin can relatively selectively reduce the abundance of *Akkermansia* and alter purine metabolism such that inosine levels decrease. Besides, the thickness of the colonic mucus layer declines in the colistin treatment group while the gut permeability is increased as assessed by serum lipopolysaccharides (LPS) levels. As a result, colistin downregulates PD-L1 expression in the testis and promotes the activation of CD4^+^ GZMB^+^ T cells. Moreover, oral supplement with inosine restores the thickness of the mucus layer in the colon and reduces serum LPS levels, leading to the recovery of PD-L1 expression and testosterone secretion in the testis. These findings provide preliminary evidence that some specific gut bacteria influence the sex hormone biosynthesis in the testis.

## MATERIALS AND METHODS

### Animals and treatments

C57BL/6N male mice (7 weeks old) were purchased from Beijing Vital River Laboratory Animal Technology Co. Ltd. All mice were maintained under specific pathogen-free conditions and performed under the guidance of the Animal Protection and Use Committee of Qingdao University (QDU-AEC-2021131). After 1 week for adaptation, mice were treated with 1 mg/mL colistin (Aladdin, C114323) in sterile drinking water for 1 week. For inosine treatment, mice were given 300 mg/kg inosine by gavage each day for 1 week, and sterile PBS was used as the vehicle control. Fresh feces, cecal contents, serum samples, testis, and colon tissues were collected before the end of the experiment. For surgical orchiectomy, male mice underwent sham operation or bilateral orchidectomy via scrotal incision under pentobarbital anesthesia as previously described (28) and were allowed to recover for 5 weeks. Then, these mice were treated with 1 mg/mL colistin for 1 week.

### ELISA

Serum samples were collected and examined for the concentration of testosterone (Abbkine, KET0001) or LPS (Cloud-Clone Crop, HEB526Ge) using commercially available enzyme-linked immunosorbent assay (ELISA) kits per the manufacturer’s instructions.

### Flow cytometric analysis

Testis tissues were collected, mechanically dissociated, and then digested in 1640 medium containing 1 mg/mL Collagenase IV/0.15 mg/mL DNase I for 40 min at 37°C. After passing through a nylon mesh, cells were blocked with anti-CD16/32 antibody (eBioscience) and then stained with Fixable Viability Dye eFluor 660 (BioLegend, 65-0864-14) to identify live and dead cells. For intracellular cytokine staining, single-cell suspensions were stimulated with a cell activation cocktail (with Brefeldin A) (BioLegend, 423304) at 37°C for 4 h and immobilized and permeated using the Foxp3/Transcription Factor Staining Buffer Kit (eBioscience). Then, the cells were stained with conjugated antibodies to CD45, CD4, CD8, PD-L1, IFNγ, IL-4, and Granzyme B (all from BioLegend). Samples were analyzed with NovoCyte flow cytometry, and the data were analyzed using FlowJo software (V.10.6.2, Tree Star).

### Histology

Colon was removed, fixed with Carnoy’s fixative, embedded in paraﬃn, and sliced into 5 µM sections. The parafﬁn sections were dewaxed, dehydrated, and stained with alcian blue (Servicebio, G1049). The thickness of the colonic mucus layer was measured on 10 random visual ﬁelds per section using ImageJ software (NIH, USA).

### Fecal DNA extraction and absolute quantitative sequencing of 16S rRNA

Accurate 16S absolute quantification sequencing was performed by Genesky Biotechnologies Inc. (Shanghai, China). Briefly, total genomic DNA was extracted using the FastDNA SPIN Kit for Soil (MP Biomedicals, CA) according to the manufacturer’s instructions. Multiple spike-ins with identical conserved regions to natural 16S rRNA genes and variable regions replaced by random sequences with ~40% GC content were artificially synthesized. Then, an appropriate proportion of spike-ins mixture with known gradient copy numbers was added to the sample DNA. The V3–V4 hypervariable regions of the 16S rRNA gene and spike-ins were amplified with the primers 341F (5′-CCTACGGGNGGCWGCAG-3′) and 805R (5′-GACTACHVGGGTATCTAATCC-3′) and then sequenced using Illumina NovaSeq 6000 sequencer. After quality control (QC) with QIIME2, amplicon sequence variants (ASVs) were directly generated. ASV sequences were classified with a confidence threshold of 0.8 by a pre-trained Naive Bayes classifier trained on the RDP (version 11.5). Then, the standard curve for each sample was generated based on the read counts versus the spike-in copy number. The absolute copy number of each ASV in each sample was calculated using the read counts of the corresponding ASV.

The vegan package in R software was used to calculate the α diversity of Observed, Shannon, Simpson, Chao 1, and ACE indices. Similarity analysis of Bray-Curtis distances (ANOSIM) and permutation multivariate variation analysis (PERMANOVA) was performed to analyze the similarity between groups using vegan packages. Principal component analysis (PCA) was performed using R package ade4 and illustrated with R package made4. Linear discriminant analysis (LDA) effect size (LEfSe) analysis was used to detect differential taxa. LDA values > 2 and *P* values < 0.05 were considered significantly enriched. Co-occurrence network analysis was conducted using igraph and psych packages. Pearson’s correlations with *r* values >  0.6 and *P* values <  0.01 were used for network construction. The prediction of functional genes and the corresponding biochemical pathways were conducted in the Phylogenetic Investigation of Communities by Reconstruction of Unobserved States (PICRUSt2) package (29).

### Untargeted metabolomics

Untargeted metabolomics analysis was performed by Novogene Technology Co. Ltd. (Beijing, China). Briefly, cecal contents were individually grounded with liquid nitrogen, and the homogenate was resuspended with prechilled 80% methanol by the vortex. The samples were incubated on ice for 5 min and centrifuged at 15,000 *g*, 4°C for 20 min. The supernatant was diluted to a final concentration containing 53% methanol by liquid chromatography-mass spectrometry grade water. The samples were subsequently transferred to a fresh Eppendorf tube and centrifuged at 15,000 *g*, 4°C for 20 min. Finally, the supernatant was injected into the liquid chromatographic tandem mass spectrometric system for analysis.

Ultra-high performance liquid chromatography–tandem mass-spectrometry (UHPLC–MS/MS) analyses were performed using a Vanquish UHPLC system (Thermo Fisher, Germany) coupled with an Orbitrap Q Exactive HF mass spectrometer (Thermo Fisher, Germany). The raw data files generated by UHPLC-MS/MS were processed using the Compound Discoverer 3.1 (CD3.1, Thermo Fisher) to perform peak alignment, peak picking, and quantitation for each metabolite. The main parameters were set as follows: retention time tolerance, 0.2 min; actual mass tolerance, 5 ppm; signal intensity tolerance,

30%; signal/noise ratio, 3; and minimum intensity. After that, peak intensities were normalized to the total spectral intensity. The normalized data were used to predict the molecular formula based on additive ions, molecular ion peaks, and fragment ions. Then, peaks were matched with the mzCloud, mzVault, and MassList databases to obtain the accurate qualitative and relative quantitative results.

The metabolites were annotated using the Kyoto Encyclopedia of Genes and Genomes (KEGG) database (https://www.genome.jp/kegg/pathway.html), HMDB database (https://hmdb.ca/metabolites), and LIPIDMaps database (http://www.lipidmaps.org/). Orthogonal partial least-square discriminant analysis (OPLS-DA) was used to identify differential metabolites. In addition, univariate analysis (*t*-test) was performed to calculate the statistical significance (*P* value). The metabolites with variables important in the projection (VIP) > 1 and *P* value < 0.05 and |log2 Foldchange| > 1 were considered differential metabolites. The volcano plot and heatmap were constructed to visualize the differential metabolites using R packages ggplot2 and ComplexHeatmap, respectively. The metabolic pathways were analyzed by MetaboAnalyst 5.0 (https://www.metaboanalyst.ca/) based on the KEGG pathway enrichment.

### Statistical analysis

Statistical analyses were conducted using GraphPad Prism 9.0 (GraphPad Software Inc., USA). Unless otherwise indicated, all continuous variables were presented as means ± SEM and compared between groups using Student’s *t*-test. *P* < 0.05 was considered to be statistically significant.

## RESULTS

### Colistin downregulates PD-L1 expression and promotes the infiltration of CD4^+^ T cells in the testis

**Fig 1:**
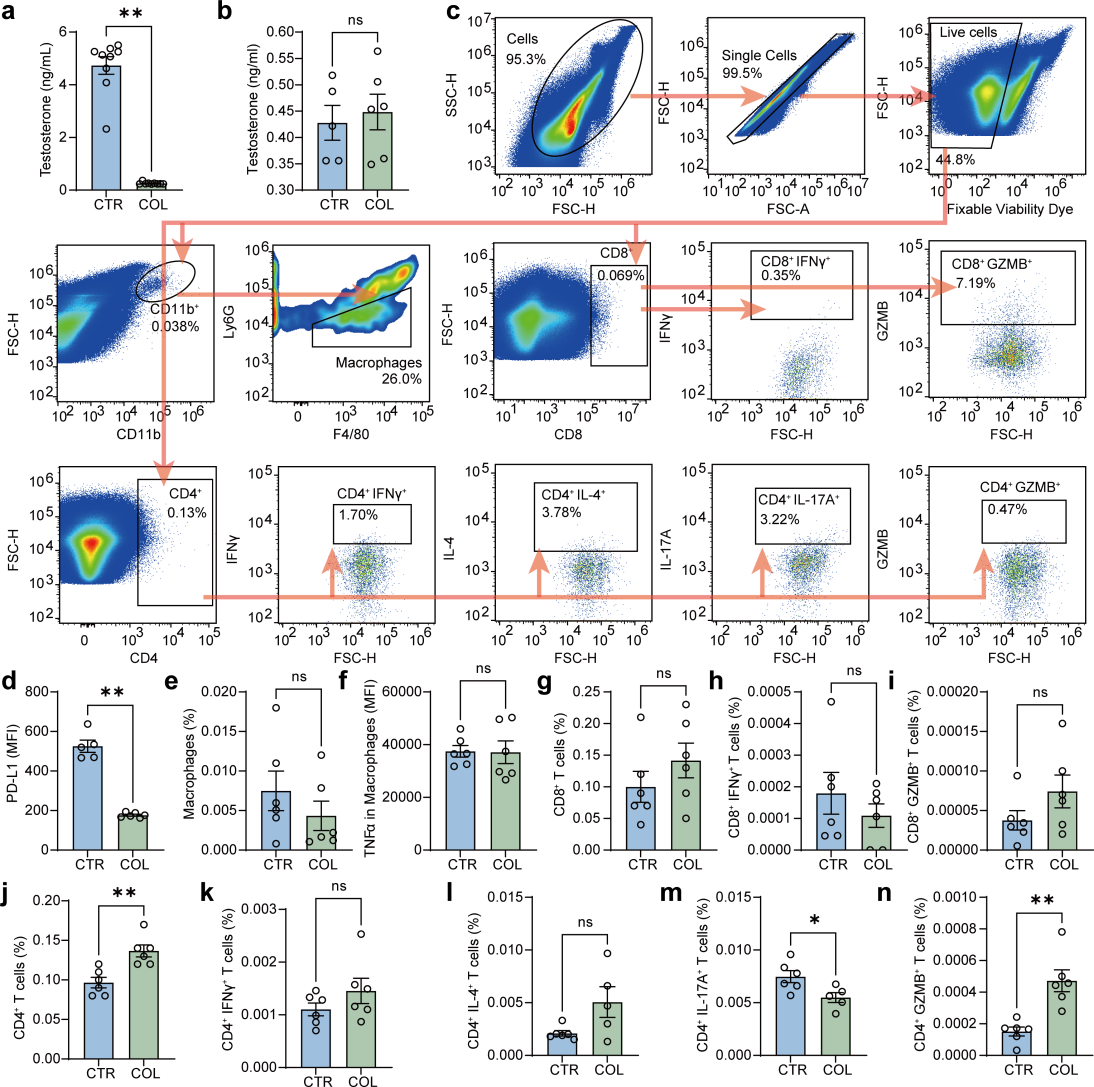
The impacts of colistin on serum testosterone levels and testicular immune microenvironment in male mice. After treatment with 1 mg/mL of colistin in sterile drinking water for 1 week, serum testosterone levels were measured by ELISA in non-castrated mice (a) or castrated mice (b). (c) Gating strategy for flow cytometry analysis. PD-L1 expression (d), infiltration of macrophages (e), TNFα expression in macrophages (f), and infiltration of CD8^+^ T cells (g), CD8^+^ IFNγ^+^ T cells (h), CD8^+^ Granzyme B^+^ T cells (i), CD4^+^ T cells (j), CD4^+^IFNγ^+^ Th1 cells (k), CD4^+^IL-4^+^ Th2 cells (l), CD4^+^IL-17^+^ Th17 cells (m), and CD4^+^ Granzyme B^+^ T cells (n) in the testis were analyzed by FACS. All data are presented as mean ± SEM. *P < 0.05, and **P < 0.01. CTR, control; COL, colistin.

In this study, we first investigated the possible effects of colistin on testosterone secretion in the testis. As shown in Fig. 1a, the serum testosterone levels significantly decreased in male mice receiving colistin treatment (1 mg/mL in sterile drinking water for 7 days) compared with mice in the control group. This was also studied in male mice undergoing bilateral orchidectomy and allowed to recover for 5 weeks to ensure that all the testosterone produced in the testis had been metabolized (30). In contrast, colistin has no apparent influence on the serum testosterone levels in male mice post-castration (Fig. 1b). These results indicated that colistin might directly impact testosterone biosynthesis in the testis. Considering that the testis is an immune‐privileged organ, the breakdown of the testicular immune privilege may contribute to impaired testosterone synthesis (31). Therefore, we next investigated the impact of colistin on PD-L1 expression in the testis and the infiltration of macrophages, which are the primary immune cell subtype present in the testis (32). As indicated in Fig. 1c through f, colistin treatment significantly downregulated PD-L1 expression in the testis while showing no apparent effects on the infiltration and activation of macrophages. Our previous study showed that anti-PD-L1 antibodies can dramatically promote the infiltration of CD4^+^ T cells and decrease testosterone secretion (22). Therefore, we next ask if there is a similar effect for colistin. As shown in Fig. 1g through i , colistin had no significant impact on the infiltration and activation of CD8^+^ T cells. In contrast, the infiltration of CD4^+^ T cells was markedly increased in the colistin treatment group (Fig. 1j). Further study showed that colistin can enhance the infiltration of CD4^+^ IFNγ^+^ Th1 and CD4^+^IL-4^+^ Th2 cells in the testis, although there is no significant difference (Fig. 1k through l). In contrast, the infiltration of CD4^+^ IL-17A^+^ Th17 cells significantly declined in the colistin treatment group (Fig. 1m), while there was a noticeable increase in the infiltration of CD4^+^ GZMB^+^ T cells (Fig. 1n). These results indicate that oral colistin administration impacts the immune microenvironment within the testis, which may contribute to the inhibition of testosterone biosynthesis in the testis.

### Colistin relatively selectively decreases the abundance of *Akkermansia*

**Fig 2:**
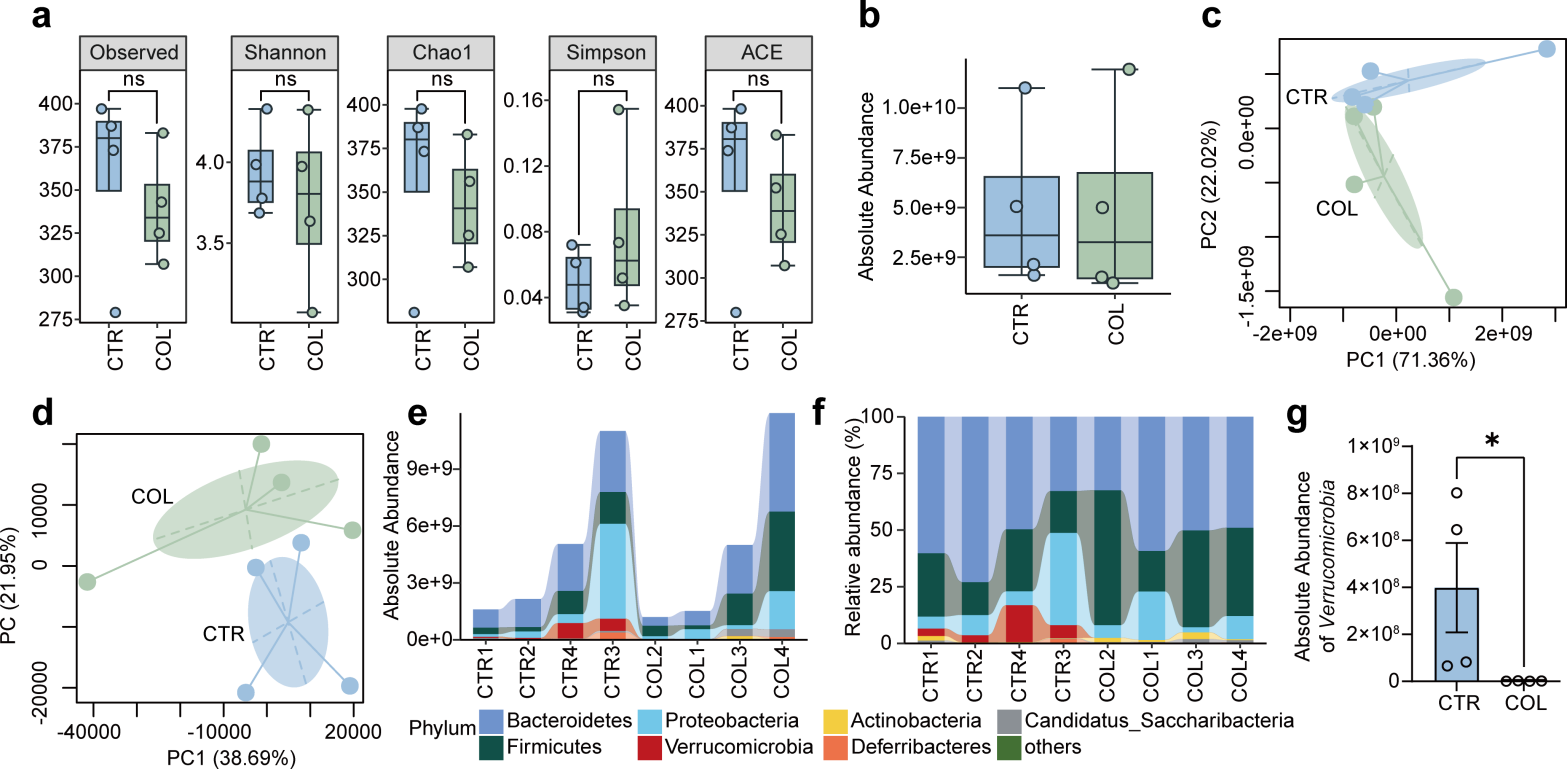
Effects of colistin on the gut microbiota composition in male mice. (a) Comparison of alpha diversity indices at the species level. (b) Comparison of the total absolute abundance. Principal component analysis of the absolute (c) and relative (d) abundances of ASVs. The absolute (e) and relative (f) composition of the gut microbiota at the phylum level. (g) Comparison of the absolute abundances of *Verrucomicrobia*, mean ± SEM, Mann-Whitney test. *P < 0.05.

Colistin is a narrow spectrum with an extremely low absorption rate from the gastrointestinal tract (33). Thus, we hypothesized that the changes in the gut microbiota’s composition may mediate colistin’s effects. To test our hypothesis, we collected the fecal samples after treatment with colistin for 7 days and performed accurate 16S absolute quantification sequencing. Consistent with its narrow antibacterial spectrum, there is no significant difference in the alpha diversity between the colistin treatment group and the control group (Fig. 2a). Similarly, the absolute abundance of the total bacterial load per gram of fecal sample showed no significant difference between the groups (Fig. 2b). The PCA based on the absolute abundance of ASVs showed apparent changes in the gut microbiota structure, although the difference was not significant (ANOSIM, *P* = 0.194; Adonis, *P* = 0.067) (Fig. 2c). The relative beta diversity analysis showed that the colistin treatment group was markedly separated from the control groups (Fig. 2d). The gut microbiota structure significantly differed between groups (ANOSIM, *P* = 0.031; Adonis, *P* = 0.03). At the phylum level, the absolute abundance of each bacterial group varied greatly between individual samples, among which both the absolute and relative abundance of *Firmicutes*, *Bacteroidetes*, and *Proteobacteria* dominated the bacterial communities (Fig. 2e and f). Remarkably, the phylum *Verrucomicrobia* disappeared in the colistin treatment group, which showed a significant difference (Fig. 2g).

**Fig 3:**
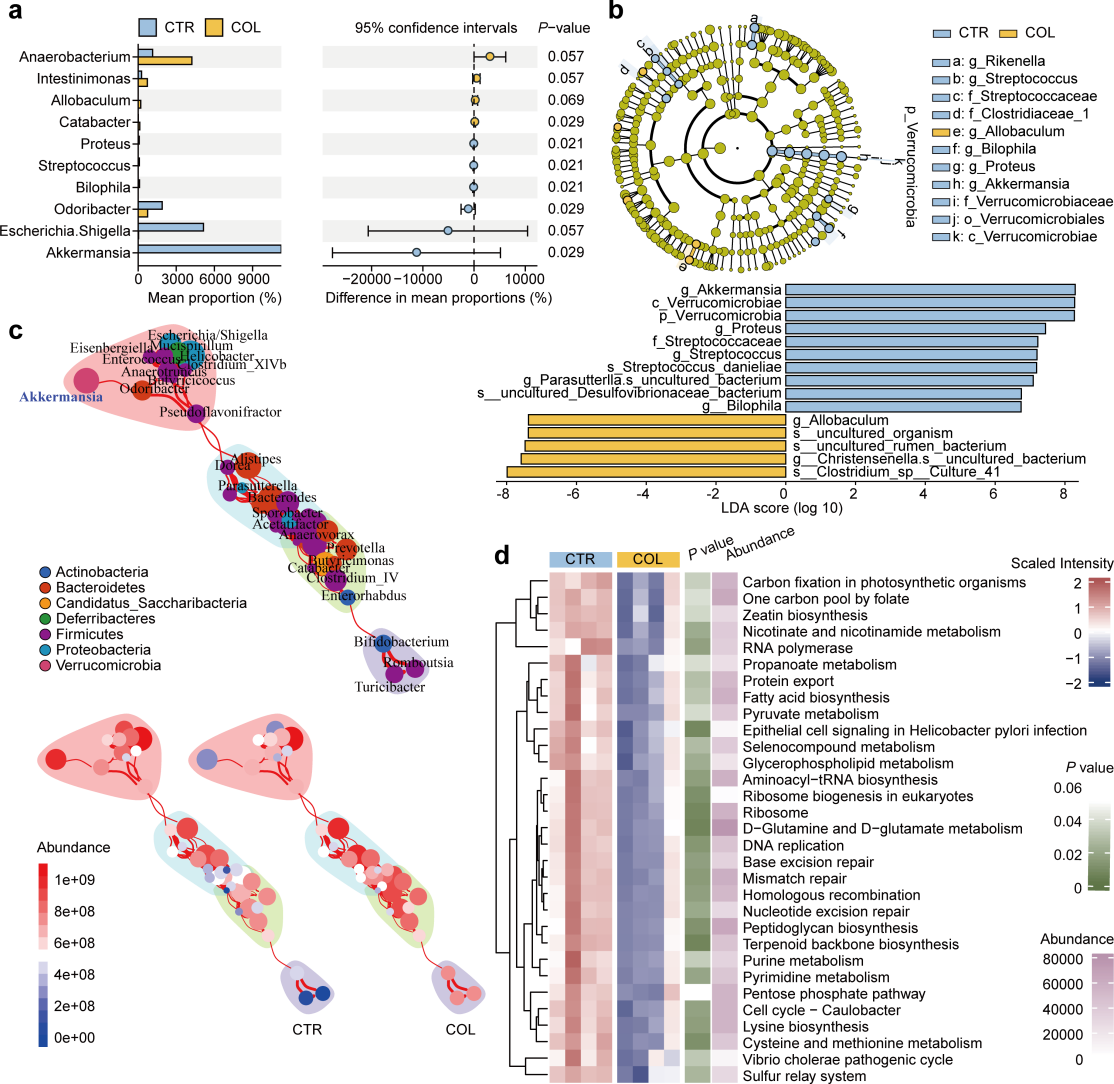
The alteration of the gut microbiota at different taxonomic levels and changes in the potential functional profiles. (a) Comparison of the gut microbiota composition between the groups by statistical analysis of taxonomic and functional profiles at the genus level. *P* values were derived from the Wilcoxon rank sum test. (b) LEfSe analysis of the gut microbiota composition. (c) Co-occurrence network graphs of the gut microbiota. (d) Heat map of differentially abundant KEGG pathways based on the PICRUSt2 analysis.

To identify all the differential species at the genus level, we then performed the Wilcoxon rank sum test and found that *Proteus*, *Streptococcus*, *Bilophila*, and *Akkermansia* significantly decreased after colistin treatment for 7 days (Fig. 3a). However, it’s worth noting that the absolute abundances of *Proteus*, *Streptococcus*, and *Bilophila* are relatively negligible compared with *Akkermansia*. We also performed LEfSe analysis to further determine the differential bacteria from the phylum to the genus level. As shown in Fig. 3b, the results indicated that phylum *Verrucomicrobia* only includes one genus, *Akkermansia*, which is lower in the colistin treatment group from the phylum to the genus level. The co-occurrence network analysis results showed that the change in *Akkermansia* is relatively independent (Fig. 3c). Besides, PICRUSt2 was used to predict the functional profiling of microbial communities based on the KEGG database. As detailed in Fig. 3d, some metabolism pathways, such as fatty acid biosynthesis, pyruvate metabolism, and purine metabolism, were significantly inhibited by colistin treatment. These results suggest that, as a narrow spectrum, colistin can selectively inhibit several particular bacterial species, especially *Akkermansia*, which may alter the metabolism of the gut microbiota.

### Colistin inhibits the purine metabolism of the gut microbiota

**Fig 4:**
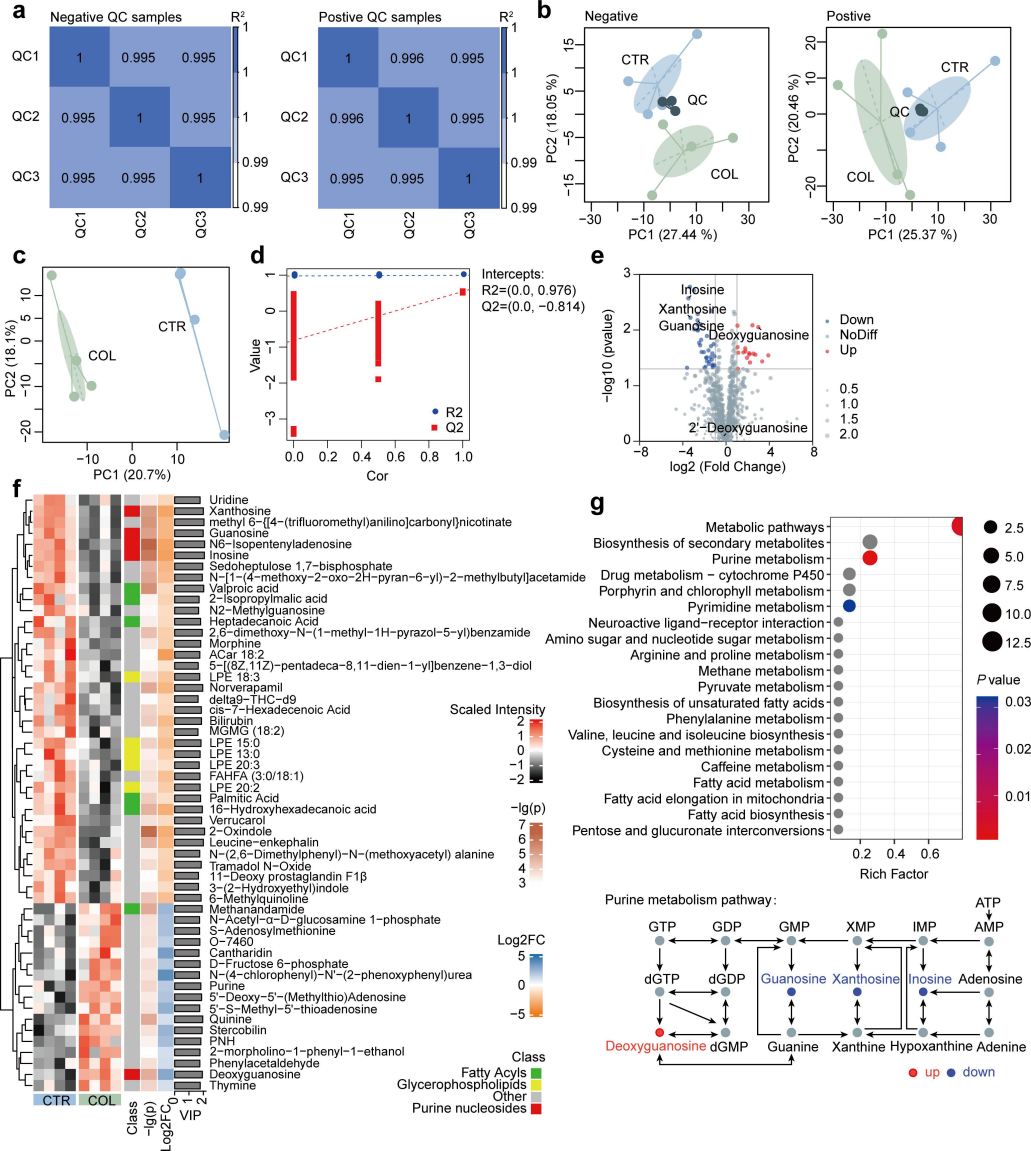
Colistin treatment changes the gut microbiota metabolism in male mice. (a) The Pearson correlation of quality control samples. (b) PCA analysis of the QC and experimental samples in both positive and negative modes. (c) OPLS-DA score plot. (d) Cross-validation plot with a permutation test repeated 200 times. The intercepts of the goodness-of-ﬁt (*R*^2^) are greater than the goodness-of-prediction (*Q*^2^), and the intercept of *Q*^2^ is less than 0, indicating no overfitting. (e) Volcanic map of the differential metabolites (red, upregulated; blue, downregulated). Student’s *t*-tests were performed for the comparison. (f) Heat map of differential metabolites. (g) KEGG pathway enrichment analysis of differential metabolites and the pathway diagram of purine metabolism derived from the KEGG database (red, upregulated; blue, downregulated).

To investigate the metabolic changes induced by colistin, untargeted metabolomics was performed on cecal contents. The Pearson’s correlation coefficients of quality control samples were high in both positive and negative modes, suggesting the reliability of our metabolomic data (*r* > 0.99) (Fig. 4a). The PCA analysis showed that the quality control samples are tightly clustered together, further confirming the data’s high quality (Fig. 4b). Besides, the colistin treatment group samples are obviously separated from the control group in both positive and negative modes, indicating the alteration of the metabolites. Then, we performed the OPLS-DA to identify the potential biomarkers that differentiate the two groups. As shown in Fig. 4c, there is a clear separation between these two groups. The robustness and reliability of the OPLS-DA classification model were validated by sevenfold cross-validation. The *R*^2^*Y* and *Q*^2^*Y* values in this OPLS-DA model were 0.994 and 0.758, respectively, demonstrating a high predictive ability. To avoid overfitting, 200 random permutation tests were performed to validate the OPLS-DA model further. As shown in Fig. 4d, the intercept of the goodness-of-ﬁt (*R*^2^) and goodness-of-prediction (*Q*^2^) were 0.976 and −0.814, respectively, indicating that the original model is reliable and not overﬁtting. Then, the VIP values were calculated in the OPLS-DA model, and a total of 54 signiﬁcantly differential metabolites were identified (Fig. 4e; VIP > 1 and *P* value < 0.05 and |log2 Foldchange| > 1). As shown in the heatmap of Fig. 4f, there are 17 upregulated and 37 downregulated metabolites in the colistin treatment group compared with the control group. Among them, purine nucleosides, fatty acyls, and glycerophospholipids were the main differential metabolite categories. KEGG pathway enrichment analysis showed that the differential metabolites were mostly enriched in the metabolic pathways of purine metabolism (Fig. 4g and h), which is in consistent with the PICRUSt2 results (Fig. 3d). Collectively, these results suggested that particular metabolic pathways are altered by colistin treatment, especially the purine metabolism, which may contribute to the inhibition of testosterone secretion in the testis.

### Supplementation of inosine promotes the recovery of the gut barrier and restores serum testosterone levels

**Fig 5:**
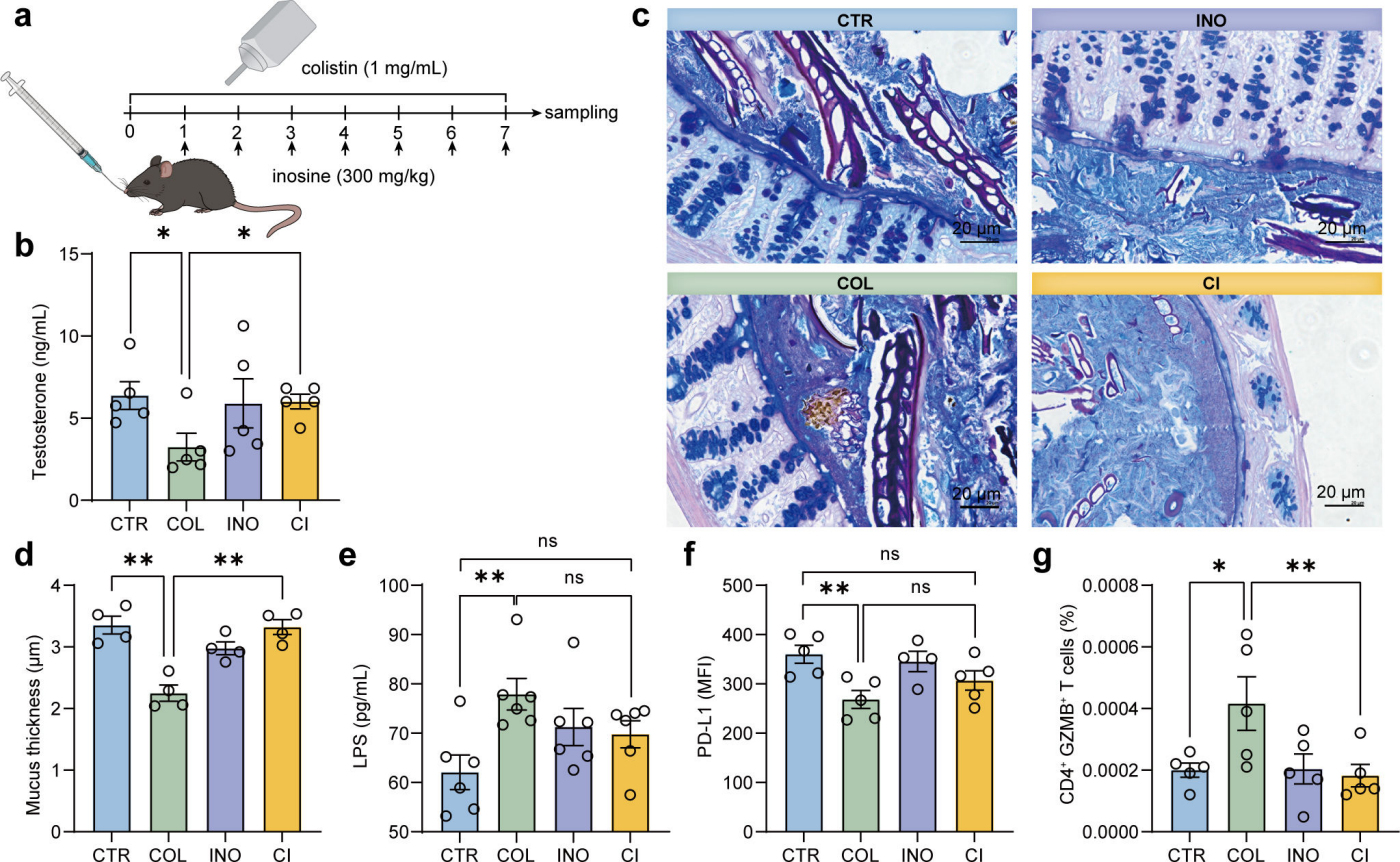
Supplementation of inosine promotes the recovery of the gut barrier and testicular immunosuppressive microenvironment and restores the serum testosterone levels in male mice. (a) Schematic diagram of the experimental design and sample collection. For inosine treatment, mice were given 300 mg/kg inosine by gavage daily for 1 week, with sterile PBS as the vehicle control. (b) Serum testosterone levels were measured by ELISA. (c) Representative images of PAS/Alcian Blue staining of the colon. Scale bars: 20 µm. (d) The thickness of the mucus layer. (e) Serum levels of LPS. PD-L1 expression (f) and infiltration of CD4^+^ Granzyme B^+^ T cells (g) in the testis were analyzed by FACS. All data are presented as mean ± SEM. *P < 0.05, and **P < 0.01. CTR, control; COL, colistin; INO, inosine; CI, colistin plus inosine.

Previous studies suggested that *Akkermansia muciniphila*, an inosine producer strain, plays a vital role in maintaining the gut barrier and is inversely correlated with serum LPS levels (34, 35). Since LPS-initiated inﬂammation has been associated with the inhibition of testosterone production in the testis (36), we next asked whether the decline of the inosine production triggered by colistin would lead to the impairment of the gut barrier and the inhibition of testosterone production. Indeed, oral supplement with inosine noticeably restored the decreased testosterone production caused by colistin treatment (Fig. 5a and b). Results of the periodic acid-Schiff and Alcian blue stains revealed that the inner mucus layer in the colon was thinner than that in the colistin treatment group (Fig. 5c and d). In addition, the serum LPS levels were significantly increased by colistin, suggesting an increased gut permeability (Fig. 5e). Interestingly, supplement with inosine could restore the mucus layer thickness and attenuate the increase in serum LPS levels (Fig. 5c through e). Further analysis showed that inosine could promote the recovery of PD-L1 expression and reduce the infiltration of CD4^+^ GZMB^+^ T cells in the testis (Fig. 5f and g). These results indicated that the decline in the inosine production mediates the inhibition of testosterone secretion in the testis, possibly by inducing the dysfunction of the gut barrier and the testicular immunosuppressive microenvironment.

## DISCUSSION

**Fig 6:**
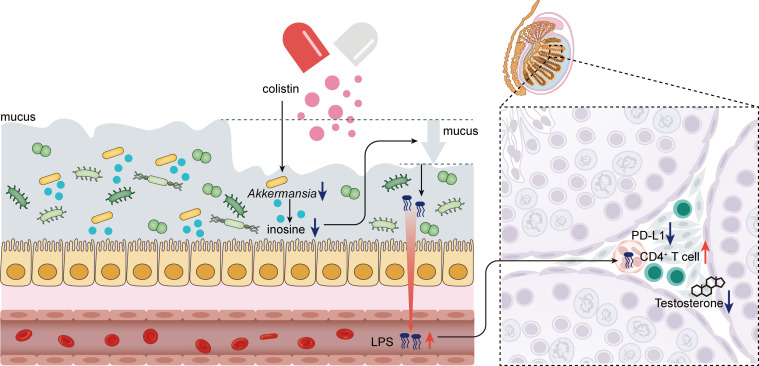
Inhibition of *Akkermansia* decreases the production of inosine in the gut, leading to the disruption of the colonic mucus layer. The abnormal serum LPS level due to the increased gut permeability may contribute to the inhibition of testosterone secretion by modulating the immune microenvironment within the testis.

Growing evidence indicated that antibiotic administration is associated with poor immune response to ICIs (37). In contrast, our previous study showed that the narrow-spectrum antibiotic colistin can significantly enhance the antitumor efficiency of anti-PD-L1 by downregulating testosterone levels in male mice (22). However, the detailed underlying mechanisms remain uninvestigated. Here, we found that colistin can significantly downregulate PD-L1 expression in the testis and enhance the infiltration of CD4^+^ T cells, suggesting an impact on the immunosuppressive microenvironment of the testis. Further investigation showed that colistin can relatively selectively reduce the abundance of *Akkermansia* and decrease the production of inosine. Oral supplement with inosine attenuates the altered thickness of the colonic mucus layer induced by colistin and restores normal serum LPS levels. Moreover, the inosine supplement promotes the recovery of PD-L1 expression and testosterone secretion in the testis. These findings reveal a new pathway for the regulation of the host’s testosterone secretion by gut microbiota (Fig. 6).

Recent studies indicated that the gut microbiota provides an essential alternative source of androgens (23). The excreted glucuronidated testosterone metabolites in the intestine can be reabsorbed via the enterohepatic circulation after deconjugation by gut bacterial β-glucuronidases (16). In young adult men, the concentration of free dihydrotestosterone in feces is more than 70-fold higher than in serum (25). In addition, some specific gut-colonizing bacteria may be capable of directly metabolizing steroid hormones by expressing steroid processing enzymes (27). Therefore, broad-spectrum antibiotic treatment can significantly downregulate the serum testosterone levels in castrated mice (23). In contrast, our results showed that the narrow-spectrum antibiotic colistin can directly affect testosterone secretion in the testis by inhibiting inosine production and damaging the mucosal barrier. Moreover, supplement with inosine promotes the recovery of the mucosal barrier and testosterone secretion. These findings suggest a new possible strategy for regulating testicular testosterone biosynthesis.

*Akkermansia muciniciphila* (*A. muciniphila*), the most important species of the *Akkermansia* genus, is emerging as a potential immune booster, especially in cancer immunotherapy (12, 38, 39). Both short-chain fatty acids (SCFAs) and inosine, the vital metabolites of *A. muciniphila*, have been proven beneficial for tumor immunotherapy (34, 40). One crucial possible mechanism for the immune regulatory function of *A. muciniphila* is the participation in the maintenance of the intestinal mucus layer (35, 41, 42). However, the underlying mechanisms for the protection of the mucus layer by the mucin-degrading bacterium *A. muciniphila* remain elusive. SCFA has been reported to be able to induce the production of mucin in goblet cells (38). Besides, low concentration of butyric acid, one of the SCFAs, could directly stimulate progesterone secretion in porcine granulosa cells (43). Thus, we cannot exclude the possible impacts of SCFAs on testosterone secretion, which deserves further study in the future. Nonetheless, in this study, we found colistin relatively selectively inhibits *Akkermansia* and decreases the production of inosine and the mucus layer thickness. Moreover, supplement with inosine promotes the recovery of the mucosal barrier and the testosterone levels. This is consistent with previous studies showing gut microbiota-derived inosine improved mucosal barrier functions by activating the PPARγ signal pathway and increasing the number of mucus-producing goblet cells (44). These findings suggested that the *Akkermansia*-derived inosine may play a critical role in maintaining the intestinal mucus layer. It is of note that the abundance of *Proteus*, *Streptococcus*, and *Bilophila* is also significantly decreased by colistin. Although the absolute abundances of these bacteria are relatively negligible, we cannot exclude their possible role in the regulation of inosine metabolism and testosterone secretion. Supplementation of *Akkermansia* should be able to promote recovery of the mucosal barrier and the testosterone levels. However, colonization of *Akkermansia* may also induce changes in the abundance of other bacteria. Germ-free mice colonized with *Akkermansia* can be used in the future to provide direct evidence for the role of *Akkermansia*.

Inosine could be released by some gut bacteria, such as *Biﬁdobacterium pseudolongum* and *Akkermansia muciniphila* (34). As a prominent source of exogenous purines, these microbiota-sourced metabolites are crucial for the maintenance of the mucous barrier integrity (45). Dysfunction of the intestinal barrier may enhance the translocation of LPS into systemic circulation leading to high circulating LPS levels (46). Considering that a single injection of a low dose of LPS is enough to decrease the Leydig cell testosterone production (47), the intestinal barrier dysfunction induced by defects in the inosine metabolism of the gut microbiota may impair the testosterone production via increasing circulating LPS levels. Indeed, our present results showed that supplementation of inosine promotes the recovery of the mucus barrier, serum LPS levels, and testosterone production. However, the detailed underlying mechanisms still need further exploration.

Tremellen has put forward the theory of gut endotoxin leading to a decline in gonadal function to explain the obesity-related male hypogonadism (36). Consistent with this hypothesis, our results showed that the defects in the intestinal mucus barrier can also increase serum LPS levels. It has been found that the intraperitoneal injection of LPS could induce dysfunction of the testis and epididymis and signiﬁcantly reduce serum testosterone levels via induction systemic inﬂammation (48, 49). Besides, LPS could directly induce pyroptosis of Leydig cells and decrease testosterone production (50). However, our results showed that colistin has no noticeable impact on macrophage infiltration and TNFα production despite an increase in the serum LPS levels, probably due to the relatively low amplitude of LPS concentration changes induced by colistin. Instead, colistin treatment can downregulate PD-L1 expression and enhance the infiltration of CD4^+^ T cells in the testis, which are consistent with our previous results that administration of anti-PD-L1 increased CD4^+^ T cell infiltration and decreased testosterone secretion in male mice (22). These findings hinted that the moderate increase in the LPS levels may have an effect on the testicular immune microenvironment without inducing severe inﬂammation. However, future research still needs to identify the underlying mechanisms for the downregulation of PD-L1 expression and the resulting inhibition of testosterone biosynthesis.

The intestinal mucus secreted by goblet cells forms an essential barrier between the host and gut microbiota, preventing bacterial invasion and inflammation (51). Multiple factors, such as infection (41), high-fat diet (52), and inflammatory bowel disease (53), may result in the dysfunction of the mucus barrier. Interestingly, Paone and Cani reported an about sixfold decline in the colonic mucus layer’s thickness in aged mice compared with young mice (54). Moreover, long-term supplementation of *Akkermansia muciniphila* ameliorates the age-related decline in colonic mucus thickness (42). Since the defects in the intestinal mucus layer can interfere with testosterone secretion in the testis, administration of *Akkermansia muciniphila* or the key metabolite inosine may represent a new promising way for the supplement of testosterone in aged men, which deserves future investigation.

In summary, we found that colistin, as a narrow-spectrum antibiotic, can relatively selectively decrease the abundance of *Akkermansia*. As a result, colistin treatment downregulates serum testosterone levels in male mice by inhibiting inosine production, disrupting the immunosuppressive microenvironment in the testis. Supplement with inosine can restore testosterone secretion probably by prompting the recovery of the intestinal mucus barrier and serum LPS levels. All these findings reveal a new pathway for the regulation of the host’s sex hormone levels by gut microbiota.

## Data Availability

The fecal bacterial 16S rRNA sequencing data set has been deposited in the SRA database of NCBI with the Accession ID of SRP469110. All other data needed to evaluate the conclusions in this paper are presented in the paper. Additional data related to this paper may be requested from the authors.
